# Enhanced light-harvesting by plasmonic hollow gold nanospheres for photovoltaic performance

**DOI:** 10.1098/rsos.171350

**Published:** 2018-01-24

**Authors:** Hao Ding, Jindian Lv, Huaping Wu, Guozhong Chai, Aiping Liu

**Affiliations:** 1 Key Laboratory of E&M, Ministry of Education, Zhejiang University of Technology, Hangzhou 310014, People's Republic of China; 2 Center for Optoelectronics Materials and Devices, Zhejiang Sci-Tech University, Hangzhou 310018, People's Republic of China; 3 State Key Laboratory of Nonlinear Mechanics, Institute of Mechanics, Chinese Academy of Sciences, Beijing 100190, People's Republic of China

**Keywords:** hollow gold nanospheres, TiO_2_ nanorod, surface plasmon resonance, photochemical performance, solar cell

## Abstract

A ‘sandwich'-structured TiO_2_NR/HGN/CdS photoanode was successfully fabricated by the electrophoretic deposition of hollow gold nanospheres (HGNs) on the surface of TiO_2_ nanorods (NRs). The HGNs presented a wide surface plasmon resonance character in the visible region from 540 to 630 nm, and further acted as the scatter elements and light energy ‘antennas' to trap the local-field light near the TiO_2_NR/CdS layer, resulting in the increase of the light harvesting. An outstanding enhancement in the photochemical behaviour of TiO_2_NR/HGN/CdS photoanodes was attained by the contribution of HGNs in increasing the light absorption and the number of electron-hole pairs of photosensitive semiconductors. The optimized photochemical performance of TiO_2_NR/HGN/CdS photoanodes by using plasmonic HGNs demonstrated their potential application in energy conversion devices.

## 1. Introduction

Recently, quantum dot-sensitized solar cells (QDSSCs) have attracted increasing interest and are considered as a potential alternative to silicon-based photovoltaic devices [1–3]. Titanium dioxide (TiO_2_) with a wide-band gap (approx. 3.2 eV) is the most commonly used photoanode material in solar cells, which absorbs only ultraviolent light [4–6]. Generally, the light harvesting of QDSSCs is mainly determined by the quantum dot (QD) sensitizers, such as CdS [7–9], CdSe [10–12], CdTe [12] and PbS [13]. Narrow-bandgap QDs have high absorption cross section and absorb light in the visible to infrared (IR) range, which can be combined with wide-bandgap TiO_2_ semiconductors to form a heterostructure and allow excellent charge transport [14–22].

In the past decades, considerable efforts to augment photovoltaic performance in QDSSCs by perfecting the counter electrode [23], electrolytes [24] and sensitizers [7–13] have been implemented. Noble metal nanoparticles (NPs), especially Au and Ag nanocrystals, have been given significant attention for their ability to couple incident photons in the visible to near IR region through the creation of surface plasmon resonances (SPRs) [25,26]. Plasmonic nanostructures can act as subwavelength scattering elements to fold light into a thin absorber, and also be used antennas to concentrate light energy surrounding them, which finally act as the electron sink for facilitating charge separation [27]. The plasmonic properties can be controlled by the type of the metal, size, shape, surrounding dielectric medium, distance between neighbouring objects and configuration of their ensemble [28–30]. For instance, Zhang *et al*. decorated CdSe-TiO_2_ photoanode with Au NPs and found that the photocurrent increased to 85% and the photoconversion efficiency increased to 167% due to the electron sink of Au NPs [31]. Bardhan *et al*. demonstrated enhanced light harvesting in DSSCs with silica-coated Au nanocubes, resulting in a power conversion efficiency of 7.8% [32]. We also clarified the enhanced photovoltaic performance of CdS- or CdSe-sensitized photoanodes with plasmonic Au NPs as the efficient light scattering elements to couple and trap the sunlight [33,34].

**Figure 1.**
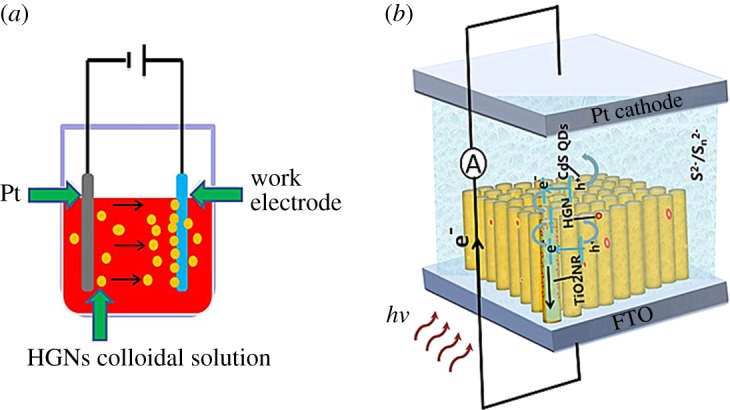
(*a*) Schematic of surface decoration strategy of TiO_2_ nanorods electrodes based on electrophoretic deposition in the HGNs colloidal solution. The DC voltage was 35 V. (*b*) Schematic of separation and transfer of electron-hole pairs in CdS QD-sensitized TiO_2_ solar cell with plasmonic HGNs.

Compared with the solid Au NPs, the hollow gold nanoparticles (HGNs) have stronger plasmon resonance and wider plasmonic tuning range [35,36]. However, as far as we know, there is no investigation of photovoltaic performance of DSSCs by employing HGNs as light scattering centres. In this paper, we introduce the HGNs into the TiO_2_NR (nanorod) structure by a facile electrophoretic technology (figure 1*a*). The CdS sensitizer was deposited on the TiO_2_NR/HGN surface by the successive ionic layer adsorption and reaction (SILAR) to construct the ‘sandwich'-structured photoanodes (figure 1*b*). The physico-chemical properties of TiO_2_NR/HGN/CdS photoanodes and their photovoltaic performances had been systematically studied, which would enable us to better understand SPR behaviour of HGNs in a photovoltaic material with adjustable light absorption and photovoltaic response.

## 2. Material and methods

### 2.1. Materials and reagents

FTO-coated glass (F : SnO_2_, resistivity of 14Ω/square and thickness of 300** **nm, Nippon Sheet Glass, Japan) was used as transparent conducting oxide substrate. Chloroauric acid (HAuCl_4_·3H_2_O, 99.99%), sodium borohydride (NaBH_4_), cadmium nitrate (Cd(NO_3_)_2_), sodium sulfide (Na_2_S), sodium sulfite (Na_2_SO_3_) and titanium butoxide (Ti(OCH_2_CH_2_CH_2_CH_3_)_4_, 99%) were purchased from Sigma-Aldrich Co. Trihydrate sodium citrate (TCD, C_6_H_5_O_3_N^.^2H_2_O, purity 99%), cobalt chloride hexahydrate (CoCl_2_^·^6H_2_O), hydrochloric acid (HCl, 36%), ethanol (C_2_H_6_O), methanol (CH_4_O) and acetone (C_3_H_6_O) were supplied from Eagle Chemical Reagent Co. Ltd. All chemicals were of analytical grade and used as received. All aqueous solutions were prepared using deionized (D.I.) water with a resistivity of 18.2 MΩ cm prepared by Millipore Q purification system.

### 2.2. Preparation of citrate-stabilized hollow gold nanospheres

The citrate-stabilized HGNs are fabricated by using Co NPs as sacrificial templates with starting material of HAuCl_4_ [37,38], as reported by Zhang *et al.* [39]. In brief, 75 ml of D.I. water was firstly placed into a three-necked Erlenmeyer bulb with 0.1** **mol l^−1^ TCD in a 35°C water bath, and the solution was deoxygenated by ultrapure argon. After 30** **min, 100 μl of CoCl_2_ (0.4** **mol l^−1^) was added into the flask under vigorous magnetic stirring (2500** **r.p.m.), and a certain amount of fresh NaBH_4_ (1** **mol l^−1^) was added into the mixture solution. The colour of solution was changed from pale pink to brown within tens of seconds, indicating the reduction of Co^2+^ to cobalt NPs. After the hydrolysis reaction of NaBH_4_ was finished, 30 ml Co NP colloidal solution was transferred to aqueous solution of HAuCl_4_ (0.6** **mmol l^−1^, 10 ml) under continuous stirring. Finally, the as-prepared HGNs solution was centrifuged twice and re-dispersed into D.I. water. The solid gold nanoparticles (SGNs) used as a reference were synthesized by the classic Frens' method [35].

### 2.3. Preparation of photoanodes for photovoltaic cells

The FTO glass substrates (10 mm × 20** **mm) were cleaned ultrasonically with acetone, ethanol and D.I. water, respectively, and placed into the Teflon-liner at an angle against the wall. Then 0.32 ml of Ti(OCH_2_CH_2_CH_2_CH_3_)_4_ was slowly added into the HCl solution within 5** **min and transferred into the Teflon-lined stainless steel autoclave (50 ml) for TiO_2_NR growing at 150°C for 12** **h. For the electrophoretic deposition of HGNs on the TiO_2_NR anode (figure 1*b*), a two-electrode system was used with the Pt foil as the cathode and the separation distance of two electrodes was 15** **mm. A DC voltage of 35** **V was applied for 3–13** **min to infuse the HGNs on the TiO_2_NR surface in a 10 ml of HGN dispersion solution (0.04** **mM) while ceaselessly stirred. Then the electrodes were dried at 60°C to improve the contact between HGNs and TiO_2_NR. The TiO_2_NR/HGN samples were further sensitized *in situ* with CdS by SILAR deposition by immersing in 0.1** **mol l^−1^ Cd(NO_3_)_2_ ethanol solution and 0.1** **mol l^−1^ Na_2_S methanol : D.I. water solution (1 : 1 by volume) several times. Various quantities of HGNs and CdS were easily controlled by changing the deposition time of HGNs and SILAR cycles for CdS to optimize the photovoltaic performance of photoanodes. In addition, the FTO/TiO_2_NR/CdS photoanode without HGNs and the FTO/TiO_2_NR/SGN/CdS photoanode were also prepared as control to explore the effect of HGNs in the photoelectrochemical behaviours of photoanodes.

### 2.4. Characterization

The morphologies of different samples were characterized by transmission electron microscopy (TEM, Philips CM 300 FEG) at 200** **kV, and scanning electron microscopy (SEM, Hitachi S4800, Japan) with an energy-dispersive X-ray (EDX) spectrometer. X-ray diffraction (XRD) pattern of sample was determined by a diffractometer (Bruker AXS D8) with Cu Kα radiation (*λ *= 0.15418** **nm) at an accelerating voltage of 40** **kV and applied current of 40** **mA. The extinction spectra of samples were measured by the UV–vis spectrophotometer (Hitachi U3900).

Photoelectrochemical performances of samples were performed on a CHI 630D electrochemical system (Chenhua Instruments, Shanghai, China). The system consisted of three electrodes and a transparent quartz cell, which was filled with Na_2_S (0.2** **mol l^−1^) and Na_2_SO_3_ (0.2** **mol l^−1^) electrolyte (25 ml). A Pt foil was used as a counter electrode with Ag/AgCl/KCl as a reference electrode. All the samples (10 mm × 20** **mm) with impregnated area of 1.5 cm^2^ were employed as working electrodes. The curves of photovoltage and photocurrent versus times were recorded by the electrochemical system using a 150 W Xe lamp (filtered, *λ *≥ 300** **nm) as the light source. The illumination intensity near the sample surface was about 100** **mW cm^−2^. Incident photon conversion efficiency (IPCE) was measured on an IPCE measurement system (Solar Cell Scan 100, Zolix) with a 150 W Xe lamp aligned into a monchromator scanning. All measurements were carried out in ambient air and at room temperature without encapsulation.

## 3. Results and discussion

### 3.1. Microstructures of TiO_2_NR/HGN/CdS photoanode

**Figure 2.**
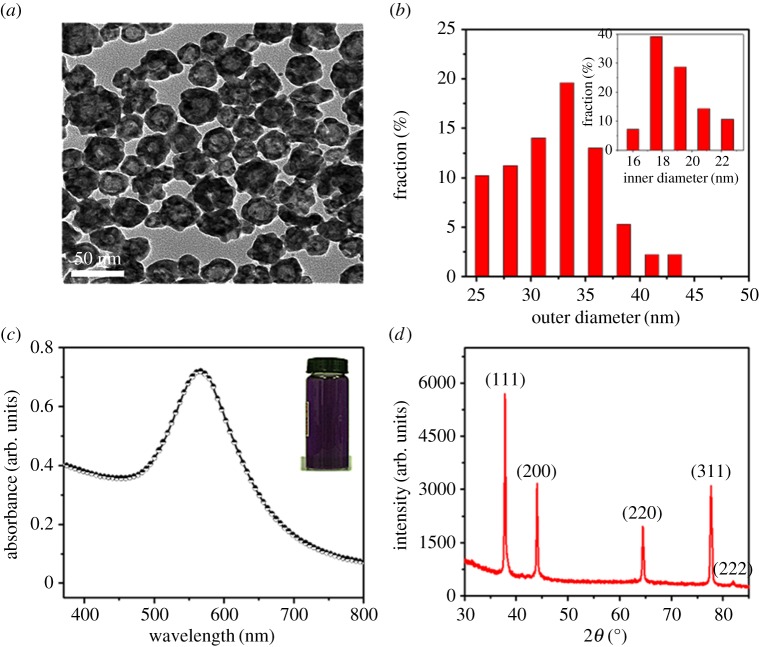
(*a*) TEM image, (*b*) size distribution histograms, (*c*) UV–vis absorption spectrum and (*d*) XRD spectrum of HGNs.

The TEM image of concentrated HGNs is shown in figure 2*a*. The strong contrast difference with a bright centre and a much darker edge confirms their hollow architecture, and their average outer diameter and inner one are about 33 ± 2.7** **nm and 19 ± 2.3** **nm, respectively, as observed from the size distribution histograms (figure 2*b*). Figure 2*c* displays the SPR absorption band centred at 575** **nm, which corresponds to HGNs [35,39,40]. The XRD pattern of the as-prepared HGNs on the glass substrate (figure 2*d*) shows four diffraction peaks at (2*θ*) 38.0°, 44.2°, 64.6° and 77.8°, respectively, which correspond to (111), (200), (220) and (311) crystallographic planes [41], confirming the face-centred cubic gold (JCPDS, 04-0784).

**Figure 3.**
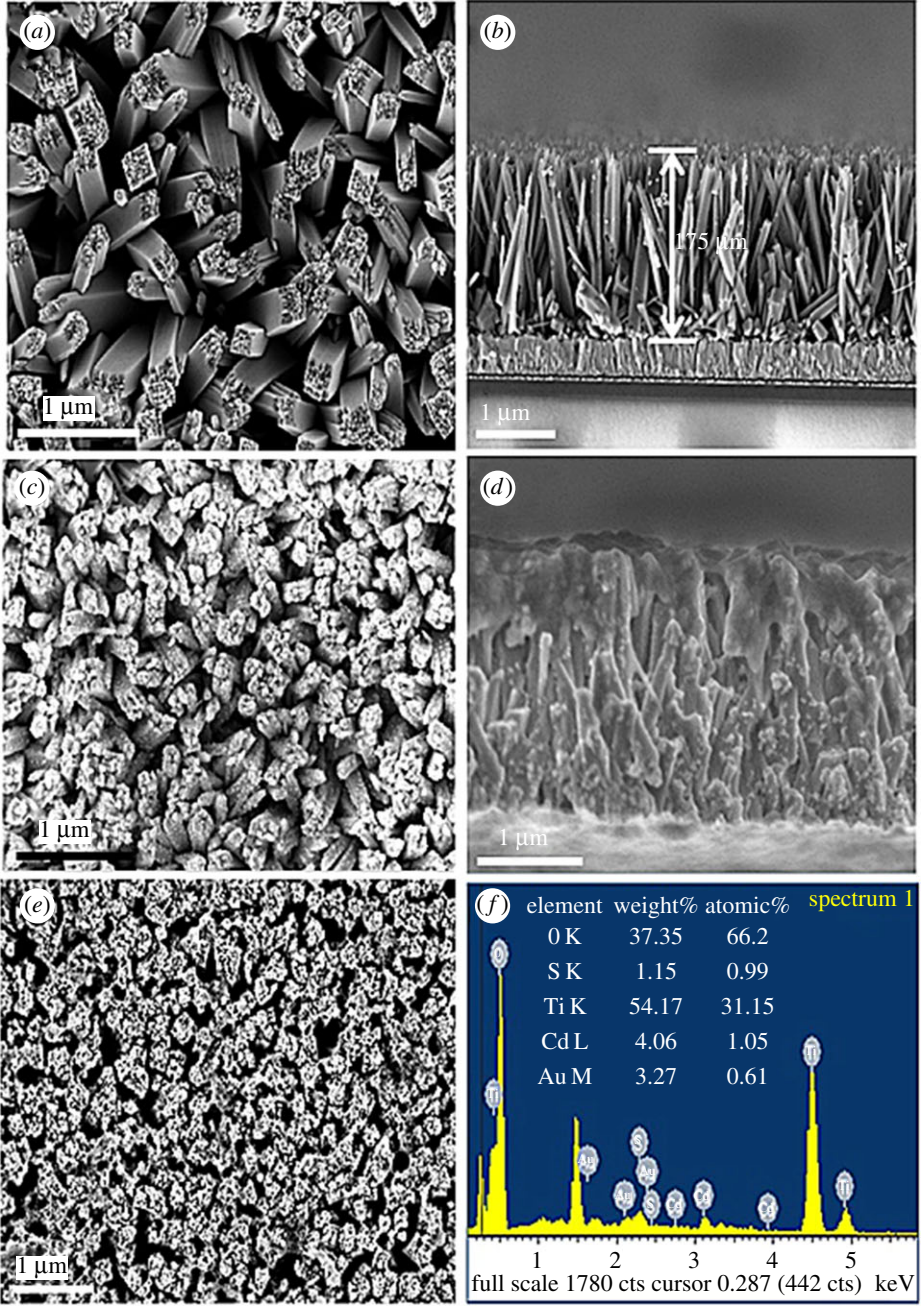
SEM images of (*a*) TiO_2_NR, (*c*) TiO_2_NR/HGN and (*e*) TiO_2_NR/HGN/CdS hybrids. Cross-sectional SEM micrographs of (*b*) TiO_2_NR/HGN and (*d*) TiO_2_NR/HGN/CdS hybrids. The electrophoretic deposition time of HGNs was 7 min, and the number of SILAR cycles applied to deposit CdS was 7. (*f*) EDS result of TiO_2_NR/HGN/CdS hybrid. The electrophoretic deposition time of HGNs was 9 min, and the number of SILAR cycles applied to deposit CdS was 7.

**Figure 4.**
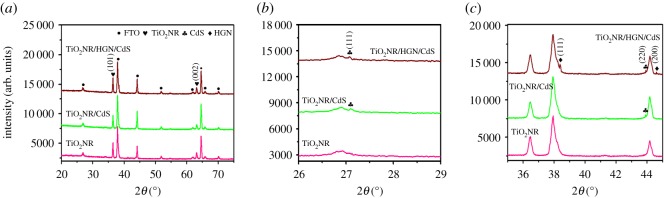
(*a*) XRD patterns of TiO_2_NR, TiO_2_NR/CdS and TiO_2_NR/HGN/CdS. Detailed XRD patterns of these three samples in the range (2*θ*) (*b*) from 26° to 29° and (*c*) from 36° to 45°. The electrophoretic deposition time of HGNs was 13 min, and the number of SILAR cycles applied to deposit CdS was 7.

**Figure 5.**
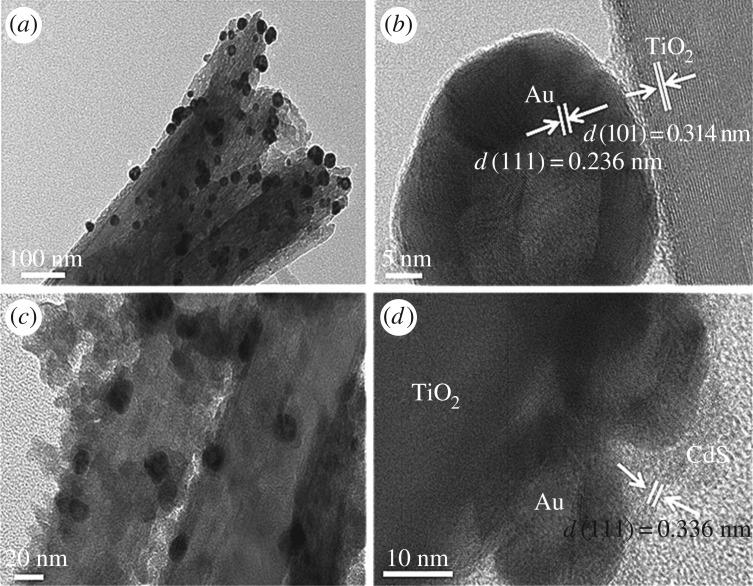
TEM images of (*a*) TiO_2_NR/HGN and (*c*) TiO_2_NR/HGN/CdS hybrids. High-resolution TEM images of (*b*) TiO_2_NR/HGN and (*d*) TiO_2_NR/HGN/CdS hybrids.

The SEM micrograph in figure 3*a* presents well-aligned TiO_2_NRs uniformly grown on the FTO substrates. The cross-sectional SEM image of TiO_2_NR with HGNs decorated in figure 3*b* indicates that the length of TiO_2_NR is about 1.75 µm. The HGNs distributed on the TiO_2_NRs surface are brighter than TiO_2_ (figure 3*b,c*). From the cross-sectional and surface SEM images of TiO_2_NR/HGN/CdS sample, we can see that many CdS QDs are well adsorbed onto the side walls of TiO_2_NR/HGN via SILAR. The EDS result shows the existence of TiO_2_, CdS and gold (figure 3*f*). The XRD peaks at (2*θ*) 36.0° and 62.7° (figure 4*a*) correspond to (101) and (002) planes of rutile structure of TiO_2_ [42] (JCPDS, 21-1276), respectively. The XRD peaks at (2*θ*) 26.6° and 43.7° are assigned to the (111) and (220) planes of cubic CdS structure (JCPDS, 65-2887) in figure 4*b,c*. The crystallographic peaks of HGNs are indistinguishable in TiO_2_NR/HGN/CdS sample due to the low content of HGNs (figure 3*d*). Furthermore, the TEM and high-resolution TEM images for TiO_2_NR/HGN and TiO_2_NR/HGN/CdS were also recorded (figure 5). The (111) facet of gold (lattice fringe spacing of 0.236** **nm) and (101) facet of TiO_2_NR (lattice fringe spacing of 0.314** **nm) are easily distinguished from the high-resolution TEM image (figure 5*b*), and the (111) crystal face of CdS with the lattice fringe spacing of 0.336** **nm is also shown in figure 5*d*.

### 3.2. Photovoltaic performance of TiO_2_NR/HGN/CdS photoanode

**Figure 6.**
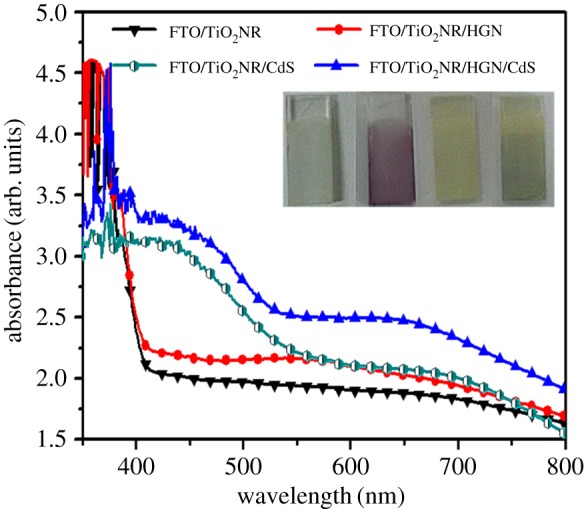
UV–vis absorption spectra and photographs of TiO_2_NR, TiO_2_NR/HGN, TiO_2_NR/HGN/CdS and TiO_2_NR/CdS hybrids. The electrophoretic deposition time of HGNs was 13 min, and the number of SILAR cycles applied to deposit CdS was 7.

**Figure 7.**
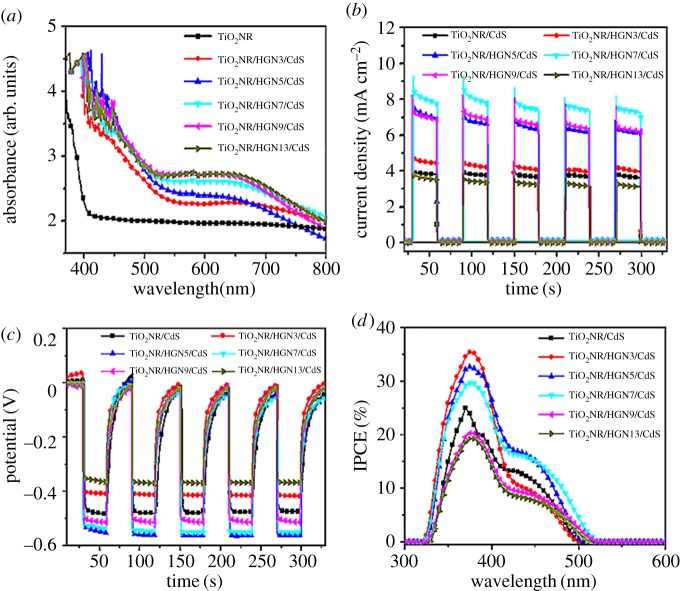
(*a*) UV–vis absorption spectra, (*b*) photocurrent density, (*c*) open-circuit voltage and (*d*) IPCE of TiO_2_NR/HGN/CdS hybrids with varied amounts of HGNs conducted by varied electrophoretic deposition times. The number of SILAR cycles applied to deposit CdS was 4.

Figure 6 displays the UV–vis absorption spectra of different photoanodes. The absorption of bare TiO_2_NR is featureless above 380** **nm, but TiO_2_NR/HGN shows an obvious absorption in the visible region between 550 and 700** **nm with respect to TiO_2_NR, which is attributed to the plasmon resonance absorption peak arising from HGNs [35,43]. As compared to the HGNs colloid in D.I. water (figure 2*c*), the plasmon band of HGNs for TiO_2_NR/HGN is red-shifted and broadened owing to the high refractive index of rutile TiO_2_ (2.55–2.76) surrounding HGNs. Since the plasmon resonance frequency of HGNs is sensitively dependent on the surrounding matter, the local electromagnetic field enhancement around the HGNs might cause the red shift of absorption and increase light absorption of the surrounding photosensitizer [34]. This means that the optical feature of TiO_2_NR/HGN is a synergistic effect between TiO_2_NR and HGNs. The broad peak from 380 to 480** **nm corresponds to the absorption of CdS QDs. Compared with TiO_2_NR/CdS, there is a significant absorption enhancement of TiO_2_NR/HGN/CdS photoanode in the region from 550 to 750** **nm, which is attributed to the behaviour of HGNs. When considering the TiO_2_NR/HGN and TiO_2_NR/HGN/CdS photoanodes, the obvious red shift of the plasmon band of HGNs could be attributed to the HGNs-mediated near-field enhancement effect and near-field coupling with photosensitive semiconductor [34]. Mahmoud's study indicated that HGNs possessed stronger plasmonic electromagnetic field and wider plasmon resonant wavelengths than the special SGNs with same size via the finite difference time domain (FDTD) calculations, which hinted that the HGNs might be more suitable as the scatter centre to concentrate photons and enhance the light absorption when compared to the SGNs [44]. To explore the light-harvesting capabilities of HGNs, the optimum amount of the HGNs was found by varying the density of HGNs on the TiO_2_NR surface with the electrophoretic deposition time from 3 to 13** **min (the number of SILAR cycles applied to deposit CdS was 4). It can be seen that as the amount of HGNs in the photoanodes increases (from 0.6 to 3.3 wt% obtained from EDS results), the absorption of TiO_2_NR/HGN/CdS in the visible region between 500 and 700** **nm is intensified by the impact of SPR absorption peak arising from HGNs when the electrophoresis times of HGNs increases from 3 to 9** **min (figure 7*a*), and tends to stabilize with the electrophoresis times rising up to 13** **min. Besides, we observe a slight red shift in the optical absorption of TiO_2_NR/HGN/CdS, which may be attributed to an increase in HGNs clusters at a certain high amounts [45].

Furthermore, the photocurrent and photovoltage of TiO_2_NR/HGN/CdS photoanodes were examined by constructing a photoelectrochemical cell system with the Ag/AgCl/KCl as a reference electrode and platinum foil as a counter electrode in the Na_2_S/Na_2_SO_3_ electrolyte (0.2** **mol l^−1^). The current densities in all samples are almost zero without photoillumination (figure 7*b*). Compared to TiO_2_NR/CdS, the TiO_2_NR/HGN/CdS samples show superior photoelectrochemical properties by introducing HGNs. The photocurrent responses of the TiO_2_NR/HGN/CdS photoanodes are strongly dependent on the deposition time of HGNs, in which the photocurrent of TiO_2_NR/HGN/CdS photoanode presents a first increased and then decreased tendency with the maximum obtained at the electrophoresis times of 7** **min (TiO_2_NR/HGN7/CdS with 2.7** **wt% HGNs obtained from EDS result). The photovoltage also shows a similar variation trend with the maximum obtained for the TiO_2_NR/HGN5/CdS or TiO_2_NR/HGN7/CdS system (figure 7*c*). The schematic representation of separation and transfer of electron-hole pairs in CdS QD-sensitized TiO_2_ solar cell with plasmonic HGNs is demonstrated in figure 1*b*. Upon photoillumination, CdS QDs will preferentially absorb incident light to produce photogenerated electron-hole pairs. The electrons are then injected into the conduction band of TiO_2_NR and further rapidly transported to the FTO substrate, while the holes are got by the electrolyte, resulting in the photocurrent in the circuit and open-circuit voltage. When the plasmonic HGNs are introduced into the TiO_2_NR/CdS system, the HGNs would absorb the incident light, scatter and trap it into the CdS film by multiple scattering, causing an increase of effective optical path length in the cell (figure 1*b*) [33,46]. This could favour the strong local-field enhancement around the HGNs and near-field coupling with the CdS semiconductor, increasing the absorption of CdS and the number of electron-hole pairs [27]. However, the intensity of localized electric field is weakened with the reduced ‘hot spots' when the HGNs greatly overlap [33] (as the recombination centres of the electrons and holes), resulting in the dramatic drop in the photovoltaic response for the TiO_2_NR/HGN9/CdS and TiO_2_NR/HGN13/CdS systems. The IPCE result measured in the 300–600** **nm wavelength range (figure 7*d*) follows a similar trend to that observed in the photocurrent responses spectra (figure 7*b*). The IPCE spectral characteristics correspond well to the absorbance spectra of the CdS-sensitized photoanodes. It can be observed that HGN-decorated TiO_2_NR/CdS photoanodes show improved photoactivity from 380 to 530** **nm compared with the TiO_2_NR/CdS, especially in the visible region from 450 to 530** **nm due to the main contribution from HGNs. These results demonstrate that excitation of the HGNs SPR is responsible for the improved photoelectrochemical properties of TiO_2_NR/HGN/CdS structures. When using the SGNs with similar size to the HGNs, the photoelectrochemical properties of TiO_2_NR/SGN/CdS structures are obviously inferior to the TiO_2_NR/HGN/CdS structures, demonstrating the preponderance of HGNs in light harvesting.

**Figure 8.**
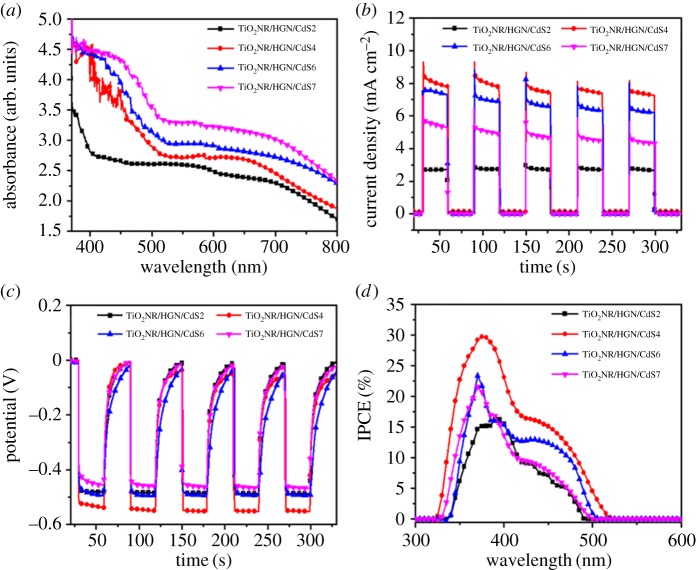
(*a*) UV–vis absorption spectra, (*b*) photocurrent density, (*c*) open-circuit voltage and (*d*) IPCE of TiO_2_NR/HGN/CdS hybrids with different numbers of SILAR cycles applied to deposit CdS. The electrophoretic deposition time of HGNs was 7 min.

For further optimize the photovoltaic performance of TiO_2_NR/HGN/CdS photoanode, the amount of CdS QDs was adjusted by changing the SILAR cycles. The UV–vis absorption spectra of TiO_2_NR/HGN/CdS photoanodes are enhanced by increasing the number of SILAR cycles (*n* = 2, 4, 6, 7) (figure 8*a*). Furthermore, the absorbance spectra related to plasmon mode of HGNs in the range from 550 to 700** **nm show a red shift with the increasing number of SILAR cycles, indicating the increased coupling and synergistic effect between HGNs and CdS. Additionally, the photocurrent density and open-circuit voltage increase with the number of SILAR cycles up to 4 (figure 8*b,c*). However, those parameters further decrease when the number of SILAR cycles is up to 6 and 7. This phenomenon may be attributed to the following reasons: firstly, the excessive SILAR cycles would lead to the conglomeration of CdS QDs, resulting in the poor charge injection efficiency due to the large QDs; secondly, the aggregation of CdS QDs may form excessive grain boundaries between CdS NPs, which would act as potential barriers and enhance the recombination possibilities of electron-hole pairs, leading to a decrease in photovoltaic behaviours [34,47,48]; thirdly, the conglomeration of excess CdS crystal nucleus may hinder the diffusion of the electrolyte into the TiO_2_NR/HGN/CdS photoanode, which also limits the efficiency of charge separation and charge extraction, as shown in the IPCE responses (figure 8*d*).

## 4. Conclusion

In summary, the ‘sandwich'-structured TiO_2_NR/HGN/CdS photoanode was fabricated by using plasmonic HGNs as light concentrators to effectively enhance photovoltaic performance. The light scattering and trapping of plasmonic HGNs and the near-field coupling of plasmonic HGNs with the photosensitive layers increase the absorption and the number of photoproduced electron-hole pairs of semiconductor. The TiO_2_NR/HGN/CdS photoanode with optimized design presents an obvious predominance in the improvement of photovoltaic performances such as photocurrent, open-circuit voltage and incident photon-to-current conversion efficiency when compared to the TiO_2_NR/CdS one. The plasmonic light-trapping concept would have potential applications in QDs-sensitized film solar cells and related energy conversion devices.

## Data accessibility

The details of the research materials pertaining to this work have been provided in the manuscript itself.
